
JOURNAL INFORMATION
==============================
NLM Title Abbreviation: Innov Surg Sci
Journal ID: Innov Surg Sci
NLM Journal ID: 101708165
Journal ID: iss

Innovative Surgical Sciences

EISSN: 2364-7485
Publisher: De Gruyter

ARTICLE INFORMATION
==============================
PMCID: PMC11005951
DOI: 10.1515/iss-2024-2001
Article ID: iss-2024-2001
Article version: 1
Subjects: Article

Abstracts DGT

Electronic publication date: 2024 Apr 9
Publication date: 2024 Apr
Volume: 9
Issue: Suppl1
First page: 1
Last page: 11
Copyright: © 2024 by Walter de Gruyter Berlin/Boston
Copyright year: 2024
Copyright holder: De Gruyter
License: This work is licensed under the Creative Commons Attribution 4.0 International License.
License URL: https://creativecommons.org/licenses/by/4.0/

Page count: 11

==============================

Abstracts – DCK 2024 – 141st Congress of the German Society of Surgery – Leipzig, April 24–26 • DOI 10.1515/iss-2024-2001
Innov Surg Sci 2024; 9 (Special Suppl 1): s1–s11

**The Goddard emphysema score does not predict postoperative air-leak in anatomic pulmonary segmentectomies**
ID: 125

Lisann Rheinhold[1 2], Thomas Galetin[1 2], Rachel Klamer[1], Ahmed Alkhatam[1], Aris Koryllos[1 3]

[1]*Florence-Nightingale-Hospital, Thoracic surgery, Düsseldorf, Deutschland*, [2]*University Witten/Herdecke, Thoracic surgery, Witten, Deutschland*, [3]*Universität Witten/Herdecke, Pulmologie, Witten, Deutschland*

**Background:**
Considering increasing patient age, comorbidities and number of small lung tumours in the context of lung cancer screening, segmental resections become more frequent and important. Software for preoperative 3D visualisation is increasingly used in surgical planning and also allows quantification of pulmonary emphysema (Goddard-score). The inevitable intraparenchymatous resection in segmentectomies increases the potential for postoperative air-leakage. The aim is to evaluate the Goddard score and other parameters as predictors of postoperative fistula duration.

**Materials and methods:**
All patients with anatomical segment resections between 01.01.2022 and 30.06.2023 were analysed. They were included if segmentectomy was intended to be performed by VATS and if a recent, sufficient preoperative CT was available for emphysema analysis. The Goddard-Score was determined on the preoperative CT using Fuji 3D software. Primary endpoint was postoperative air-leakage duration.

**Results:**
Of 144 patients, 130 fulfilled the inclusion criteria; mean age 68±9 years, BMI 26±6kg/m², packyears 34±28, FEV1 80±18%, DLCO 61±17%, median Goddard-Score 3 [0;12]. 2% were pre-radiated, 8% pre-operated. A mean of 1.7 segments was removed. 40% of the operations were complex segment resections. The parenchyma was removed with stapler only in 87%, partially with stapler in 10% and without stapler in 3%. Adhesiolysis was performed in 39%. Mean operative time was 146±53min, postoperative length of stay 7.5±4.1 days, air-leakage duration 4.1±4.9 days. 15.4% had a prolonged air-leak over 7 days. 6% of patients were discharged with an indwelled chest tube.
The Goddard-Score does not correlate with air-leakage duration (r=-0.04). Multiple linear regression for air-leakage duration (continuous outcome) reveals low BMI (p<0.01), need for adhesiolysis (p=0.02), packyears (p<0.05), immunosuppression (p=0.04), and duration of surgery (p<0.01) as significant predictors.
The logistic regression for prolonged fistula duration over 7 days (dichotomous outcome) yields significant Odd's ratios for the need for adhesiolysis (OR=5.44 [1.44, 20.55], p=0.01), lower BMI (OR=0.69 [0.57, 0.84], p<0.01) and longer surgery duration (OR=1.02 [1.00, 1.03], p<0.01), whereby surgery duration has a statistically but not clinically significant influence on the occurrence of prolonged fistula duration. Immunosuppression (OR 7.35 [0.97, 55.57], p=0.05), Goddard-Score (OR 0.82 [0.67, 1.01], p=0.06), packyears (OR 1.02 [1.00, 1.04], p=0.08), and complex segmentectomies (OR 0.53 [0.14, 1.97], p=0.34) do not reach statistical significance.

**Conclusion:**
The Goddard-Score alone is not suitable for predicting prolonged air-leakage after anatomical segmentectomy. In this population, the strongest independent predictors were adhesiolysis, BMI, and duration of surgery.

Open Access. © 2024 The Author(s), published by De Gruyter. This work is licensed under the Creative Commons Attribution 4.0 International License.

Abstracts – DCK 2024 – 141st Congress of the German Society of Surgery – Leipzig, April 24–26 • DOI 10.1515/iss-2024-2001
Innov Surg Sci 2024; 9 (Special Suppl 1): s1–s11

**Survival differences by gender for resected small cell lung cancer**
ID: 166

Romina Rösch[1], Laura Klotz[1], Lena Brendel[1], Raffaella Griffo[1], Petros Christopoulos[2], Thomas Muley[3], Hauke Winter[1]

[1]*Thoraxklinik Heidelberg, Thoracic Surgery, Heidelberg, Deutschland*, [2]*Thoraxklinik Heidelberg, Thoracic Oncology, Heidelberg, Deutschland*, [3]*Translational Lung Research Center Heidelberg (DZL), Thoraxklinik Heidelberg, Heidelberg, Deutschland*

**Background:**
Small cell lung cancer (SCLC) accounts for approximately 15% of all lung cancer cases. Of these 30% are localized tumors and only 5% of those patients are eligible for surgical treatment, according to national and international recommendations. SCLC is known for having very poor outcome in survival analyses, despite multiple treatment applications. Surgery can play a role for highly selected patients.
Studies already indicating a more favorable survival in women with SCLC. But the prognostic significance of gender has not been fully investigated in patients after surgical resection within a multimodal treatment concept. Therefore, we investigated whether gender influences survival after resection of SCLC.

**Materials and methods:**
This retrospective analysis between 07/2000 and 10/2021 included clinical data of 163 consecutive patients with SCLC treated by surgical resection within a multimodal treatment concept. The surgical procedure, perioperative management, Perioperative outcomes, Overall Survival (OAS) and survival by tumor stage were identified and analyzed.

**Results:**
Of the 163 patients, 92(56.4%) had "very limited", 65(39.9%) "limited" and 6(3.7%) "extensive" disease. The distribution of tumor stage, as well as ECOG score, were almost equal in women and men. The mean age of both populations at the time of surgery was 62.6±10.3 (female) and 62.6±3.1 (male) years. Overall, 54(33.8%) of patients were female. The time from diagnosis to surgical intervention was 24.3±40.2d for women and 18.1±40.4d for men.
There was no significant difference regarding the method of resection (anatomical resection in 82%). The median follow-up was 33.4m with a follow-up rate of 93.3%. The 90d survival rate was 96.3% female and 93.4%male, respectively. OAS was significantly higher in women (59.1vs.21.7m). Survival by tumor stage was also significantly higher in women.

**Conclusion:**
Our results showed that there are significant gender differences in favor of the female gender especially in OAS and in the survival by stage - in surgically treated SCLC patients. In the perspective of an increasingly individualized tumor therapy, gender should be given special attention, therefore we consider further and detailed investigations necessary to explain and address these differences.

Abstracts – DCK 2024 – 141st Congress of the German Society of Surgery – Leipzig, April 24–26 • DOI 10.1515/iss-2024-2001
Innov Surg Sci 2024; 9 (Special Suppl 1): s1–s11

**Short-term outcomes after extended lung cancer resection after neoadjuvant immunochemotherapy.**
ID: 875

Laura Sieber[1], Stephan Trainer[1], Mesut Mese[1], Stefan Sponholz[1]

[1]*Agaplesion Markus Krankenhaus, Klinik für Thoraxchirurgie, Frankfurt am Main, Deutschland*

**Background:**
The number of inductive immunochemotherapy (IO+CTx) in patients with lung cancer and higher-grade tumor stages is increasing. Actually the impact of IO+CTx on extended lung resections is unclear. The goal of this study was to analyze the short-term outcomes after extended lung cancer resections.

**Materials and methods:**
We retrospectively analyzed our patients with extended lung cancer resections after IO+CTx between 2020-2023. In total, 17 patients were included. Descriptive statistical analysis included the characteristics of the patients, tumors, and short-term outcomes.

**Results:**
In total, 8 female and 9 male patients with a median age of 62 years (range 52-71 years) could be included in the study. 16 patients with non-small cell lung carcinoma and one patient with small cell lung carcinoma were included in the study. Majority of the patients (n = 13) received immunochemotherapy with Pembrolizumab. In total, 7 sleeve resections (1 sleeve resection, 1 sleeve resection with combined chestwall resection, 1 bilobectomy with y-sleeve-resection, 1 double-sleeve-lobectomy and 1 double-sleeve-lobectomy with bilobectomy with combined chest wall resection, 2 y-sleeve-resections), 4 lobectomies with chestwall resection, 1 bilobectomy, and 1 bilobectomy with chestwall-resection were performed. R0 resection rate was 100%. Postoperative morbidity rate was 52.9 % (n = 9). The most common observed postoperative complication was cardiac arrhythmia in 5 patients. Two patients needed revision surgery. Observed 30-day mortality was 0%. The rates of complete and major pathologic response were 35.3 % ( n = 6) and 17.6% (n = 3), respectively.

**Conclusion:**
Although the number of included patients is small, the study shows that extended resections after immunochemotherapy are feasible with a good outcome. In total, a high rate of R0 resection is achievable.

Abstracts – DCK 2024 – 141st Congress of the German Society of Surgery – Leipzig, April 24–26 • DOI 10.1515/iss-2024-2001
Innov Surg Sci 2024; 9 (Special Suppl 1): s1–s11

**Indications and outcome of pulmonary lobectomy and total pneumonectomy under ECMO Support**
ID: 19

Michael Schweigert[1], Thomas Puehler[2], Ana Beatriz Almeida[1], Attila Dubecz[3], Patrick Kellner[4], Stephan Ensminger[2], Carla Nau[4], Tobias Keck[1]

[1]*Universitätsklinikum Schleswig-Holstein, Campus Lübeck, Klinik für Chirurgie, Lübeck, Deutschland*, [2]*Universitätsklinikum Schleswig-Holstein, Campus Lübeck, Klinik für Herz- und thorakale Gefäßchirurgie, Lübeck, Deutschland*, [3]*Klinikum Nürnberg, Klinik für Allgmein-, Viszeral- und Thoraxchirurgie, Nürnberg, Deutschland*, [4]*Universitätsklinikum Schleswig-Holstein, Campus Lübeck, Klinik für Anästhesiologie und Intensivmedizin, Lübeck, Deutschland*

**Background:**
The application of ECMO in general thoracic surgery has become more common during recent years. While there are several reports on mainly minor surgical procedures and perioperative indications of ECMO, there is a scarcity of information regarding indications and outcome of major anatomical lung resections under ECMO support.

**Materials and methods:**
At a German university hospital all patients, who underwent pulmonary lobectomy or pneumonectomy under ECMO support, were identified from a prospectively collected database. Indications, ECMO setting and overall results were analyzed. Study period was 01/2018 to 06/2023. All procedures were performed by the same team of thoracic surgeons.

**Results:**
There was a total of 27 patients (9 female) with a median age of 59 years (IQR 11.5 years) and a median Charlson score of comorbidity of 1 (IQR 2). Indications for surgery were lung cancer (5), pulmonary abscess formation (20) and aspergilloma with aorto-pulmonary fistula (2). Priority level was either elective (4) or emergency surgery (23). It is noteworthy, that there was a considerable number of elective patients (8) with marginal functional operability who were completely prepared for ECMO but were actually able to undergo surgery without ECMO. Operative procedures were pulmonary lobectomy (23) and total pneumonectomy (4). ECMO setting was VV-ECMO (24) or VVA-ECMO (3). Mean ECMO days were 6.65 (1-23 days), mean ICU days 24.3 ± 11.9 and mean LOS 31.3 days ± 17.2. Successful weaning from ECMO was achieved in 21 patients. In-hospital mortality was 7/27 and additional 90-day-mortality was 1. There was no significant difference in mortality between lobectomy and pneumonectomy (0/4 vs. 7/23; OR 0.24, 95% CI: 0.01 to 5.14; p=0.36). Perioperative sepsis was associated with higher odds for fatal outcome (7/19 vs. 0/8; OR 10.20, 95% CI: 0.51-203.32, p=0.13).

**Conclusion:**
ECMO is an excellent tool to overcome marginal functional operability in elective patients with technical resectable lung cancer and to overcome temporal functional inoperability in emergency patients with conditions requiring pulmonary lobectomy or pneumonectomy. In the elective setting not only the actual ECMO cases but many more with very borderline functional operability, who would otherwise not be considered for surgery, benefit from the readiness to use ECMO and get the chance to receive operative treatment with curative intent.

Abstracts – DCK 2024 – 141st Congress of the German Society of Surgery – Leipzig, April 24–26 • DOI 10.1515/iss-2024-2001
Innov Surg Sci 2024; 9 (Special Suppl 1): s1–s11

**Transthoracic Robot-assisted minimal-invasive diaphragmatic rupture repair: a case report**
ID: 397

Raffella Griffo[1], Henrike Deißner[1], Peter Reimer[1], Hauke Winter[1], Martin Eichhorn[1]

[1]*Thoraxchirurgie, Thoraxklinik Heidelberg, Heidelberg, Deutschland*

**Background:**
Non-traumatic diaphragmatic rupture is a rare injury. Early diagnosis remains a challenge and preoperative diagnosis is difficult. We present a case of rupture of the right diaphragm with a symptomatic manifestation of hypertensive crises and dyspnea attacks.

**Materials and methods:**
A 35-year-old woman was admitted to our department due to right diaphragmatic elevation. Anamnesis revealed symptoms of shortness of breath, acute dyspnoea attacks and recurrent tachycardia with hypertensive crises. Over a period of years, the symptoms had steadily worsened, causing a significant reduction in quality of life. A right-sided diaphragmatic elevation was identified by dynamic MRI. Neither a paralysis of the diaphragm nor a diaphragmatic rupture was diagnosed. A paradoxical movement of the right hemidiaphragm was observed. Due to the pronounced clinical symptoms and following an interdisciplinary discussion, a right-sided robot-assisted thoracoscopic (RATS) diaphragmatic plication was scheduled in order to improve shortness of breath.

**Results:**
A full portal robotic 3-arm approach using a DaVinci X-System was applied. A large rupture of the right hemidiaphragm was unexpectedly observed during the surgery. The liver and the intestine were luxated into the thoracic cavity probably causing a vena cava inferior compression syndrome. The liver and small intestine were robotically repositioned and the rupture on the diaphragm was repaired with PTFE-pledget reinforced Ethibond non-resorbable sutures. In order to enable contractility of the reconstructed diaphragm additional reinforcement of the diaphragm by an alloplastic mesh was waived. The postoperative course was uneventful. A follow-up MRI 3 months after the operation showed excellent mobility of the diaphragm and clinical symptoms were disappeared.

**Conclusion:**
Non-traumatic diaphragmatic rupture should be taken into consideration in cases of radiological diaphragmatic disorders with inconclusive clinical symptoms. Clinical presentation ranges from asymptomatic cases to serious respiratory or gastrointestinal symptoms. Robot-assisted transthoracic surgery is an effective and safe minimal-invasive approach for diaphragm repair in case of diaphragmatic ruptures.

### A rare case of an exotic pleural mesothelioma – the lymphohistiocytoid variant

ID: 458

Lena Brendel[1], Michael Allgäuer[2], Florian Eichhorn[1], Helge Bischoff[3], Rajiv Shah[3], Hauke Winter[1], Laura Klotz[1]

[1]*Thoraxklinik Heidelberg gGmbH* | *Universitätsklinikum Heidelberg, Thoraxchirurgie, Heidelberg, Deutschland*, [2]*Universitätsklinikum Heidelberg Pathologisches Institut: Allgemeine Pathologie und pathologische Anatomie, Heidelberg, Deutschland*, [3]*Thoraxklinik Heidelberg gGmbH* | *Universitätsklinikum Heidelberg, Thorax-Onkologie, Heidelberg, Deutschland*

**Background:**
Malignant pleural mesothelioma is a rare tumor entity among thoracic malignancies. In addition to the three main histologic subtypes (epithelioid, sarcomatoid, biphasic), there are also rare histologic variants that present challenges even to experienced pathologists. These major subtypes have implications for the prognosis and management of patients diagnosed with this aggressive tumor.

**Materials and methods:**
A 71-year-old male patient presented in good general condition for further diagnosis of right pleural thickening without pleural effusion. In the absence of clinical symptoms and in good cardiopulmonary condition, the finding had come to attention during an externally performed diagnostic procedure using CT of the thorax to exclude pulmonary artery embolism after thrombophlebitis of the great saphenous vein on the right side. There was no known exposure to asbestos. Diagnostic video-assisted thoracoscopy revealed spherical, reddish-livid deposits on the parietal pleura and diaphragm. The visceral pleura showed no abnormalities. Representative biopsies were sent for histopathologic examination.

**Results:**
Complex histologic workup revealed the presence of pleural mesothelioma of lymphohistiocytoid subtype. Differentially, Hodgkin's lymphoma, thymic neoplasia and lymphoepithelial tumors must be considered. Characteristic were strong CK18, AE1/3, WT1, D2-40, GATA3, EMA and calretinin expressions and a conspicuous presence of the lymphoid component of the cytotoxic T cells. BAP1 showed preserved nuclear expression. CD68 and CK5/6 were found to be negative. There was no evidence of Epstein-Barr virus-related infection. Furthermore, PD-L1 expression was 80%, so immunotherapy was initiated.

**Conclusion:**
The lymphohistiocytoid variant of malignant mesothelioma is rare with only very few cases described in the literature so far. It is characterized by mesothelial cells with a histiocytelike appearance and an associated dense lymphoid infiltrate. Nevertheless, detailed immunohistochemical workup is necessary to allow accurate differentiation of subtypes for final diagnosis and thus further clinical treatment and therapeutic options.

Abstracts – DCK 2024 – 141st Congress of the German Society of Surgery – Leipzig, April 24–26 • DOI 10.1515/iss-2024-2001
Innov Surg Sci 2024; 9 (Special Suppl 1): s1–s11

**Salvage surgery of giant monophasic synovial sarcoma of the mediastinum with vena cava superior syndrome.**
ID: 548

Ali Goekkaya[1], Kanischka Ariobi[2], Mohammad Mohammad[2], Sayzana Kassahun[2], Waldemar Schreiner[2]

[1]*Universitätsklinikum Frankfurt, Thora, Frankfurt am Main, Deutschland*, [2]*Universitätsklinikum Frankfurt, Thoraxchirurgie, Frankfurt am Main, Deutschland*

**Background:**
The primary synovial sarcoma of the mediastinum is a rare tumor with an low incidence of 11% and lack of unified treatment. According to histology a monophasic, biphasic and poorly differentiated types can been distinguished. Due to the slow growing pattern most of tumors remain asymptomatic for a long time and were detected in unresectable stage combined with a compression of neighboring structures. Particularly the vena cava superior syndrome is associated with a poor prognosis. So far only four cases with monophasic mediastinal sarcoma with vena cava superior syndrome were reported, while only one patient received a complete resection after neoadjuvant chemotherapy. Three other patients demonstrated an unresectable sarcoma.

**Materials and methods:**
In a 15 year old boy has been presented in our university hospital with dyspnoea, persistent dry cough, pain in the right shoulder and constant subfebrile temperatures. Based on radiological diagnostics a giant, well-circumscribed, inhomogen tumor in the right anterior mediastinum associated with a compression of vena cava superior and complete atelectasis of the right lung were detected (Fig. 1). After a CT-guided biopsy histological analysis reveal a monophasic synovial sarcoma.

**Results:**
After 3 cycles of neoadjuvant-intended chemotherapy with ifosfamid/vincristin/actinomycin-D the tumor persists without any signs of radiologic response. Moreover a spontaneous intratumoral bleeding causing a blood transfusion and increase of inflammatory parameters in the blood analysis. Based on persisted tumor associated with vena cava superior syndrome the respectability was difficult to predict from surgical point of view. Therefore chemotherapy regime was changed to cisplatin/etoposide. According to the interdisciplinary board after completion of chemotherapy a salvage surgery was considered as the best possible treatment option in order to achieve the complete tumor resection. An extended tumor resection combined with a partial resection of pericardium, replacement of vena cava superior due to Dacron prothesis, decortication of the right lung and extended pleurectomy was performed via clamshell incision (Fig. 2, 3). The postoperative histological analysis confirmed a complete tumor resection (R0) with 95% of tumor cell necrosis demonstrating a near to complete histological response (Fig. 4, 5). Caused due to postoperative intrapleural hematoma after an extended pleurectomy and decortication of the right lung was performed 3 days later. Other hospital stay was uneventful and the patient could be dismissed on 13 day (Fig. 6). Based on goof histological response and high risk of locoregional recurrence a adjuvant chemotherapy with four cycles of ifosfamid/vincristin/actinomycin-D was performed. At 6 month follow up the patient reported a good recovery with near to regular level of physical strain. During radiological staging analysis no metastasis and no local recurrence were evident.

**Conclusion:**
Complete surgical resection is only known factor associated with long-term survival in patients with synovial sarcoma and remains a important part of aggressive multidisciplinary therapy. The extended surgery particularly in cases with vena cava superior syndrome should be expected in order to achieve the prognostically important complete resection.

Abstracts – DCK 2024 – 141st Congress of the German Society of Surgery – Leipzig, April 24–26 • DOI 10.1515/iss-2024-2001
Innov Surg Sci 2024; 9 (Special Suppl 1): s1–s11

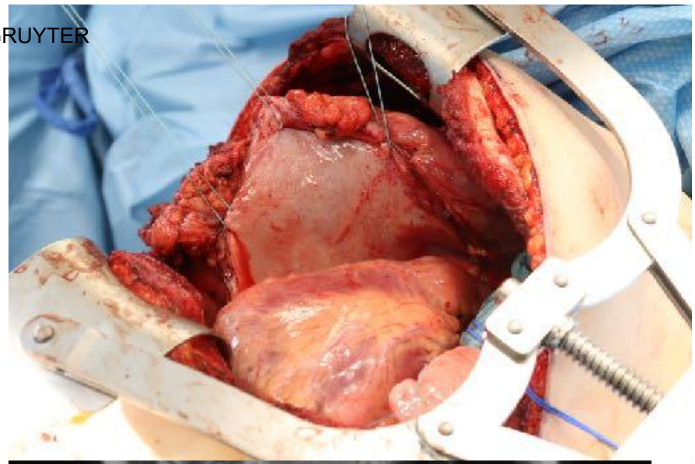

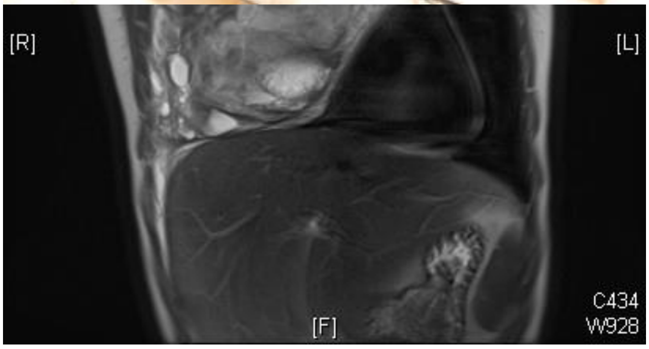

Fig. 1. Initial MRI demonstrating a massive tumor mass with compression of vena cava superior.

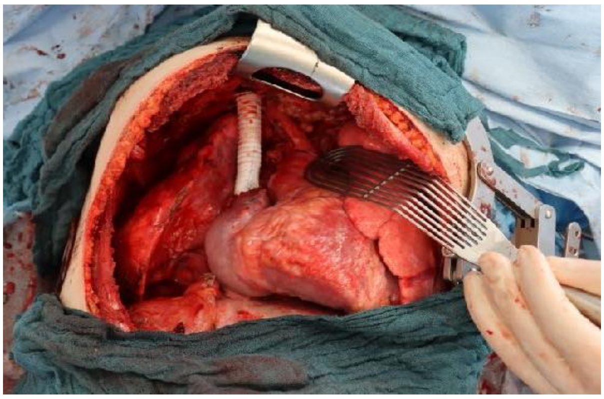

Fig. 2 and 3. Intraoperative an tumor extended resection with partial pericardectomy and vena cava superior replacement was performed.

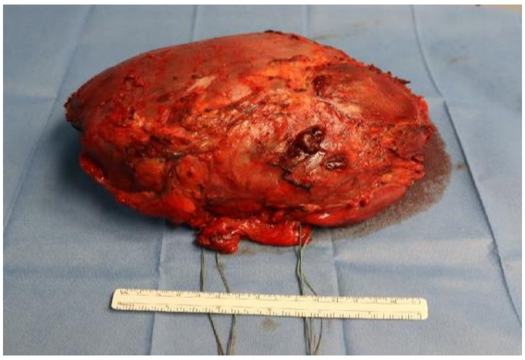

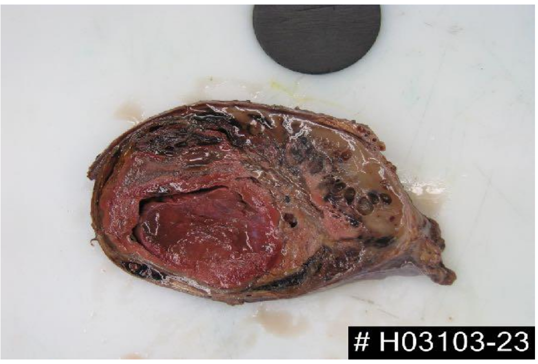

Fig. 4 and 5. The histopathology confirmed a complete resection of synovial sarcoma demonstrating a near to complete pathologic response with a 95% tumor necrosis following the preoperative chemotherapy.

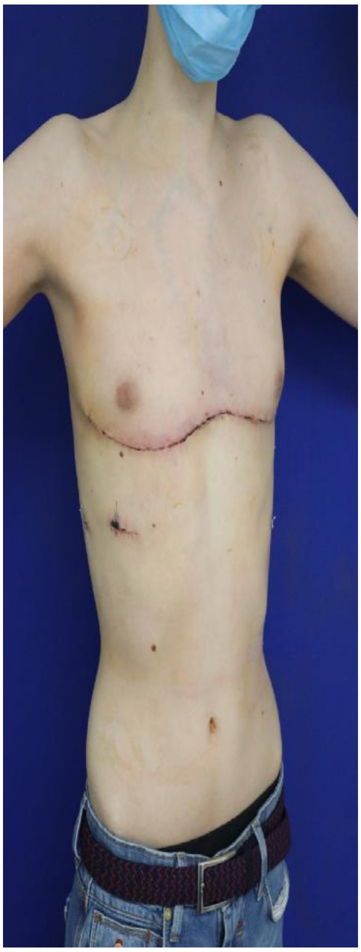

Fig. 6. The postoperative view of clamshell incision.

Abstracts – DCK 2024 – 141st Congress of the German Society of Surgery – Leipzig, April 24–26 • DOI 10.1515/iss-2024-2001
Innov Surg Sci 2024; 9 (Special Suppl 1): s1–s11

**Extralobar sequestration presenting as a bifocal, communicating posterior mediastinal tumor**
ID: 621

Ioannis Karampinis[1], Christian Galata[1], Davor Stamenovic[1], Eric Rößner[1]

[1]*Universitätsmedizin der Johannes Gutenberg-Universität Mainz, Thoraxchirurgie, Zentrum für Thoraxerkrankungen, Mainz, Deutschland*

**Background:**
A 60-year old female patient was referred for further diagnosis and treatment of a posterior mediastinal lesion. The lesion was incidentally discovered in a CT scan of the chest and abdomen, which had been performed due to an increased CA 19-9 in routine blood tests. At the time point of the referral the lesion had already been biopsied twice (CT guided and through EUS) but the histopathology was inconclusive.

**Materials and methods:**
Upon referral we repeated the CT guided biopsy and the transesophageal ultrasound. The second did not reveal any invasion of the esophagus. Both biopsies were inconclusive again. We discussed with the patient a thoracoscopic exploration in order to evaluate the extent of the disease, the exact location and its relationship to the adjacent structures.

**Results:**
The procedure was performed through a bilateral uniportal VATS approach. The lesion was originating from the right lower lobe. The lesion was mobilized from all the adjacent structures on both sides, the feeding vessel from the descending aorta was ligated and the lesion was extracted en-bloc through the left uniportal incision.

**Conclusion:**
The histopathological report revealed an extralobar pulmonary sequestration. Six weeks after surgery the CA 19-9 values had returned to normal. The transmediastinal, bilateral manifestation of the sequestration, the age of the patient and the unusual increase in the tumor markers make this altogether a very rare example of an extrapulmonary sequestration.

Abstracts – DCK 2024 – 141st Congress of the German Society of Surgery – Leipzig, April 24–26 • DOI 10.1515/iss-2024-2001
Innov Surg Sci 2024; 9 (Special Suppl 1): s1–s11

**Oligometastatic lung carcinoma or synchronous bilateral double primary lung carcinoma?**
ID: 867

Andreas Kilian[1], Stefan Sponholz[1], Mesut Mese[1], Stephan Trainer[1]

[1]*Klinik für Thoraxchirurgie, Agaplesion Markus Krankenhaus Frankfurt, Wilhelm-Eppstein-Straße 4, Frankfurt am Main, Deutschland*

**Background:**
Differentiating between a bilateral double carcinoma and an oligometastic lung carcinoma before initiating therapy is essential to define the correct treatment strategy in an interdisciplinary tumour conference. We present the case of a 62-year-old female patient with bilateral double carcinoma that was erroneously diagnosed primarily as synchronous oligometastatic lung carcinoma.

**Materials and methods:**
The patient was externally diagnosed with lung carcinoma originating from the right lower lobe of the lung with a metastasis to the left lower lung lobe and a N2 situation detected by TBNA. Immunochemotherapy with pemetrexed/carboplatin/pembrolizumab was given for a supposed stage IV A, followed by maintenance therapy with pembrolizumab. PET computed tomography showed discordant response with increased hypermetabolism and size progression on the right side and tumor regression on the left side. The patient was presented to our clinic. In case of discordant response, the interdisciplinary tumor conference recommended a bilateral resection. Initially, the right side was resected through an extended lower lobe resection with systematic lymph node dissection. Already here, a deviating pathology in the lower lobe preparation compared to the mediastinal lymph nodes became apparent, so that the suspicion of a bilateral double carcinoma was substantiated. A Y-sleeve resection of the lower lobe with systematic dissection of the lymph nodes was performed on the left side.

**Results:**
The presence of a synchronous double carcinoma with respective mediastinal N2-situation was confirmed. On the right side, there was an adenocarcinoma with mucinous differentiation and single-level N2 situation, ypT2b, ypN2 (2/43), R0, stage III A. On the left side, there was adenocarcinoma with solid, acinar and lepidic growth pattern and single-level N2 situation, ypT1c, ypN2 (5/48), R0, stage III A. Initial histopathological workup of TBNA examination from lymph node position 4 on the right showed a questionable EGFR mutation. This could not be confirmed in the postoperative workup of both surgical specimens. A formal indication for adjuvant radiotherapy due to the present N2-situation was not recommended, because there was no capsular overgrowth and due to the patient's constitution. Overall, no adjuvant therapy was performed. Since then, the patient has undergone regular follow-up with computed tomography of the thorax every six month and annual bronchoscopic sleeve checks. After 27 months of follow-up, she is still free of recurrence.

**Conclusion:**
A regular interdisciplinary exchange and critical re-evaluation of individual treatment steps and outcomes are essential in the management of complex cases. In the case of bilateral lung tumors, histologic confirmation of both sides of the tumor may be indicated to avoid premature misclassification and subsequent incorrect therapy regimen.
